# Abnormal Right Ventricular Myocardial Performance Index Is Not Associated With Outcomes in Invasively Ventilated Intensive Care Unit Patients Without Acute Respiratory Distress Syndrome—*Post hoc* Analysis of Two RCTs

**DOI:** 10.3389/fcvm.2022.830165

**Published:** 2022-05-31

**Authors:** Charalampos Pierrakos, Anna Geke Algera, Fabienne Simonis, Thomas G. V. Cherpanath, Wim K. Lagrand, Frederique Paulus, Lieuwe D. J. Bos, Marcus J. Schultz, the PReVENT– and RELAx–Investigators

**Affiliations:** ^1^Laboratory of Experimental Intensive Care and Anesthesiology (L⋅E⋅I⋅C⋅A), Department of Intensive Care, Amsterdam University Medical Centers, Amsterdam, Netherlands; ^2^Department of Intensive Care, Brugmann University Hospital, Université Libre de Bruxelles, Brussels, Belgium; ^3^Mahidol–Oxford Tropical Medicine Research Unit (MORU), Mahidol University, Bangkok, Thailand; ^4^Nuffield Department of Medicine, University of Oxford, Oxford, United Kingdom

**Keywords:** heart-lung interactions, echocardiography, hemodynamic monitoring, mechanical ventilation, mortality, successful extubation

## Abstract

**Background:**

The objective of the study was to determine the association between right ventricular (RV) myocardial performance index (MPI) and successful liberation from the ventilator and death within 28 days.

**Methods:**

*Post hoc* analysis of 2 ventilation studies in invasively ventilated patients not having ARDS. RV-MPI was collected through transthoracic echocardiography within 24–48 h from the start of invasive ventilation according to the study protocols. RV-MPI ≤ 0.54 was considered normal. The primary endpoint was successful liberation from the ventilator < 28 days; the secondary endpoint was 28-day mortality.

**Results:**

A total of 81 patients underwent transthoracic echocardiography at median 30 (24–42) h after the start of ventilation—in 73 (90%) patients, the RV-MPI could be collected. A total of 56 (77%) patients were successfully liberated from the ventilator < 28 days; A total of 22 (30%) patients had died before or at day 28. A total of 18 (25%) patients had an abnormal RV-MPI. RV-MPI was neither associated with successful liberation from the ventilator within 28 days [HR, 2.2 (95% CI 0.47–10.6); *p* = 0.31] nor with 28-day mortality [HR, 1.56 (95% CI 0.07–34.27)*; p* = 0.7].

**Conclusion:**

In invasively ventilated critically ill patients without ARDS, an abnormal RV-MPI indicative of RV dysfunction was not associated with time to liberation from invasive ventilation.

## Introduction

Acute right ventricular (RV) dysfunction is a common complication in critically ill patients and is associated with higher morbidity and mortality (1). RV function is affected by the change from negative to positive intrathoracic pressure in patients who receive invasive ventilation, by the decrease in venous return and increase in RV afterload (2). Acute RV failure in invasively ventilated patients can cause life-threatening hemodynamic instability and delay liberation from the ventilator (3, 4). Accordingly, monitoring RV function could be important for fluid optimization, vasopressor strategy, and respiratory support in these patients (3, 5).

The RV myocardial performance index (MPI) is an easy-to-obtain variable through transthoracic echocardiography (6). It is a measure for systolic and diastolic RV performance, and to a certain degree fluid status-independent (7). Right ventricular myocardial performance index (RV-MPI) has been shown to have predictive capacity for mortality in chronically ill patient, including patients with primary pulmonary hypertension (8) and patients with chronic heart failure (9). RV-MPI has also been shown to have predictive capacity for mortality in acutely ill patients, including patients after cardiac surgery (10), patients after myocardial infarction (11), patients with acute pulmonary embolism (12), and patients with sepsis (13). Whereas RV-MPI has been shown to have predictive capacity for liberation from the ventilator in critically ill ventilated patients with acute respiratory distress syndrome (ARDS) (14), it is uncertain whether it also holds prognostic capacity in critically ill ventilated patients without ARDS. To test the hypothesis that an abnormal RV function has associations with delayed extubation and higher mortality in these patients, we collected RV-MPI who underwent transthoracic echocardiography in two studies on invasive ventilation in patients without ARDS.

## Materials and Methods

### Design

This is a *post hoc* analysis of patients included in two multicenter randomized clinical trials of invasive ventilation—in one study, ventilation with a low tidal volume (V_T_) was compared with ventilation with an intermediate V_T_ (the “Protective Ventilation in Patients Without ARDS” (PReVENT) study) (15); in the other study, ventilation with lower positive end-expiratory pressure (PEEP) was compared to ventilation with higher PEEP (the “REstricted vs. Liberal positive end-expiratory pressure in patients without ARDS” (RELAx) study) (16). The results of the substudy with the PReVENT study have been published in part before (17). Echocardiography was performed as the part of two substudies that focused on the effects of the tested ventilation strategies on cardiac function and enrolled patients in only one center, the Amsterdam UMC, location “AMC,” Amsterdam, the Netherlands, from 4 November 2014 to 20 August 2017 (in the PReVENT study) and from 26 October 2017 to 17 December 2019 (in the RELAx study).

### Ethics

Ethical approval for the two parent studies (ethical committee number: 2014_075#B2014424ENG and ethical committee number 2017_074#C2017635) was provided by Medical Ethics Review Committee of AMC on 19 September 2014 and 28 June 2018. Ethical approval for the two substudies (ethical committee number W14_2992017_074 and ethical committee number #B2018435) was provided by Medical Ethics Review Committee of AMC on 4 November 2014 and 18 July 2018. Patients or relatives had to provide written informed consent before the participation in the parent study, as well as the substudy.

### Study Registration

The studies were registered at clinicaltrials.gov (NCT02153294, 3 June 2014; NCT03167580, 13 May 2017).

### Patients

The PReVENT and RELAx studies had identical inclusion and exclusion criteria and enrolled patients who received invasive ventilation shortly before and not longer than 1 h after admission to the intensive care unit (ICU) and who were expected not to be extubated within 24 h of randomization. The exclusion criteria were age < 18 years, the presence of ARDS according to the current definition of ARDS (18) known chronic obstructive pulmonary disease (COPD), pregnancy, increased and uncontrollable intracranial pressure, history of pulmonary disease, and new pulmonary thromboembolism. Patients were excluded from participation in the substudies if known poor left ventricular function, with left ventricular ejection fraction less than or equal to 30%, and severe shock, requiring norepinephrine ≥ 0.5 μg/kg/min.

### Data Collected

Patient demographics, disease severity scores, and reasons for intubation and invasive ventilation were collected at baseline. Ventilator settings and parameters, fluid status, and inotropic and vasopressor use were collected at the time of transthoracic echocardiography.

### Transthoracic Echocardiography

Transthoracic echocardiography was performed by physicians trained in cardiac ultrasound in critically ill patients using a Vivid 9 Dimension Ultrasound System (GE Healthcare, Hoevelaken, the Netherlands). Transthoracic echocardiography was performed in the supine position without any major mobilization 24–48 h after invasive ventilation initiation. A comprehensive transthoracic echocardiogram was performed, and the right and left heart were assessed using parasternal, apical, and subcostal sonographic windows. Continuous cardiac rhythm was recorded. Images and videos were stored digitally and analyzed blindly using automated function imaging software (EchoPAC^®^, GE Vingmed, Norway). For the analysis of echocardiographic variables, the median values of three or five cardiac cycles were calculated for sinus rhythm and atrial fibrillation, respectively.

Pulsatile and continuous wave Doppler was used to assess blood velocities. Tissue Doppler imaging (TDI) and motion mode (M–mode) synchronized with electrocardiogram readings were used to assess mitral and tricuspid valve annulus motion. Isovolumetric contraction time, isovolumetric relaxation time, and ejection time were calculated from the TDI trace. MPI was calculated as the ratio between the sum of the isovolumetric contraction and relaxation time to the ejection time. The two-dimensional speckle tracking for the right and left ventricles was calculated from the 4–chamber apical view after tracing the endocardial borders of the left and right ventricles. Regions of interest (ROIs) were automatically generated and manually corrected when necessary. The global longitudinal strain was calculated for the left ventricle. For the RV, the free wall was automatically divided into three segments, that is, basal, mid, and apical, and the means of the strain values were calculated for each segment.

### Outcomes

The primary outcome of this *post hoc* analysis was successful liberation from invasive ventilation within 28 days, in which successful liberation was defined as no requirement for tracheal intubation within a 48-h period following extubation and alive. The secondary outcome was the 28-day mortality.

### Statistical Analysis

The number of available patients in the substudies of the two randomized clinical trials served as the sample size for this analysis.

Demographic, clinical, echocardiographic, and outcome variables were presented as percentages for categorical variables and as medians with interquartile ranges (IQRs) for continuous variables and compared using the Mann–Whitney *U*-test or chi-square test, as appropriate. Patients were classified as having a normal or an abnormal RV–MPI based on a previously defined cutoff (RV-MPI ≤ 0.54, normal) (19).

The association of RV-MPI with outcomes was analyzed with multistate, competing risk proportional hazard models as described in the *survival* package *via* the *compete* function in R. Risks were estimated for successful extubation and mortality and compared to persistent intubation (reference category). We considered mortality and successful extubation as competing outcomes for persistent intubation. Follow-up was censored after 28 days. Patients who died and received a follow-up of less than 28 days with no events were not censored to eliminate bias through censoring by mortality. This analysis was repeated for other parameters of RV dysfunction.

Moderation of the association of RV-MPI with outcomes by V_T_ or PEEP was evaluated by adding an interaction term to the above-mentioned models. Hazard ratios (HR) with 95% confidence intervals (CI) were calculated for each outcome.

All analyses were performed in R using the R-Studio interface (R version 3.3.1)^1^ (accessed on 08/05/2022). Statistical significance was set at *p* < 0.05.

## Results

### Patients

**FIGURE 1:**
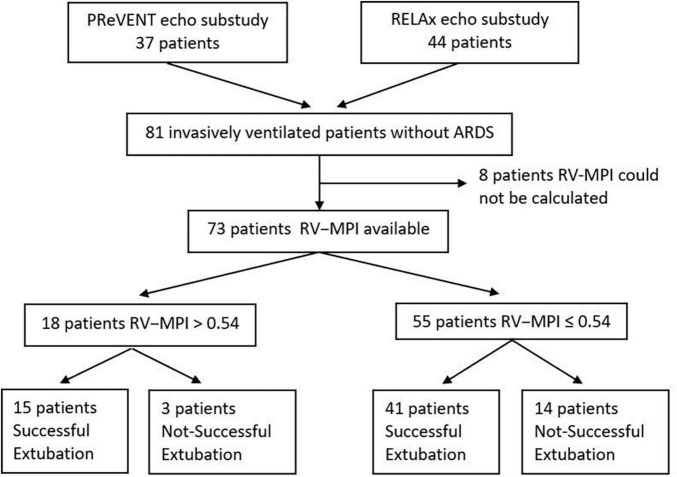
Flow chart of the patients enrolled in the study.

A total of 81 patients were enrolled in the two substudies. We excluded four patients from the cohort of patients enrolled in the substudy of the PReVENT study, because outcomes of interest were missing for these patients. Thus, we had 73 patients left for the current analysis (Figure 1). Patient characteristics are presented in Table 1. A total of eighteen patients (25%) had abnormal RV-MPI; a total of 2 patients were randomized to the high PEEP ventilation strategy, 1 patient to the low PEEP strategy, 12 patients to the high V_T_ strategy, and 3 patients to the low V_T_ strategy. A total of 55 patients (75%) had a normal RV–MPI; a total of 19 patients were randomized to the high PEEP strategy, 17 patients to the low PEEP strategy, 4 patients to the high V_T_ strategy, and 15 patients to the low V_T_ strategy. Differences in noradrenaline use or in the applied dosages did not achieve statistical significance. Echocardiography findings, including RV-MPI, are presented in Table 2.

**TABLE 1:** Demographic and clinical characteristics according to normal or abnormal right ventricular myocardial performance index (RV-MPI).

Variables	Abnormal PI (*n* = 18)	Normal RV-MPI (*n* = 55)	*P*-value
Age, years, median (IQR)	68 (56–73)	64 (54–70)	0.29
Female gender, No. (%)	5 (27)	30 (54)	0.06
Height, cm, median (IQR)	175 (170–183)	173 (168–178)	0.27
Weight, kg, median (IQR)	79 (72–88)	75 (66–71)	0.81
SOFA score, median (IQR)*	9.5 (6.5–13)	9.0 (7.0–11.0)	0.47
APACHE II score, median (IQR)*	25 (23-29)	22 (17-27)	0.34
PaO_2_/FiO_2_, median (IQR)	354 (219–375)	284 (220–370)	0.53
Medical reasons for admission, No. (%)	14 (77)	40 (72)	0.76
Reason of intubation, No. (%)			
Respiratory failure	6 (33)	11 (20)	0.17
Cardiac arrest	2 (11)	8 (15)	0.99
Depressed level of consciousness	4 (22)	12 (22)	0.73
Planned postoperative ventilation	5 (28)	19 (34)	0.98
Airway protection	1 (6)	5 (9)	0.99
Ventilatory mode, No. (%)*			
Pressure controlled ventilation	3 (17)	23 (42)	0.08
Volume controlled ventilation	5 (27)	1 (2)	<0.01
Pressure support ventilation	10 (55)	31 (56)	0.98
Vasopressor use*			
Norepinephrine, No (%)	10 (55)	19 (34)	0.09
Norepinephrine dose, μ g/kg/min, median (IQR)	0.16 (0.10–0.27)	0.11 (0.09–0.17)	0.11
Sinus rhythm, No (%)*	16 (88)	49 (89)	0.99
ICU LOS, days	9.5 (5.0-15.5)	4.5 (3.0-13.5)	0.29
Successfully extubated 28 days, No. (%)	15 (83)	41 (74)	0.53
Ventilation free days	22.5 (12.6-25.3)	18.8 (0.0-26.3)	0.66
Mortality 28 days, No. (%)	3 (16)	13 (23)	0.23
Duration of invasive ventilation, days	5.1 (2.7-10.6)	5.3 (2.3-10.6)	0.77

* At time of echocardiography.

*IQR, interquartile range; SOFA, Sequential Organ Failure Assessment; APACHE, Acute Physiology and Chronic Health Evaluation; ICU LOS, intensive care length of stay.*

**TABLE 2:** Echocardiographic variables of left and right ventricle of invasively ventilated patients examined within 48 h after mechanical ventilation initiation according to normal or abnormal right ventricular myocardial performance index (RV-MPI).

Variables	Abnormal RV-MPI (*n* = 18)	Normal RV-MPI (*n* = 55)	*P*-value
**Right ventricular function**			
***Systolic parameters***			
Myocardial performance index [n]	0.71 (0.61–0.75) [18]	0.36 (0.29–0.41) [55]	NA
Tricuspid annular plane systolic excursion (mm) [n]	16 (15–19) [18]	22 (18–26) [55]	< 0.01
Global longitudinal strain,% [n]	−12 (−18—10) [17]	−19 (−24—16) [41]	< 0.01
Isovolumetric acceleration, m/sec [n]	2.1 (1.4–2.7) [16]	3.1 (2.1–4.7) [55]	< 0.01
Systolic maximal velocity, cm/sec [n]	11 (8–12) [18]	13 (11–16) [55]	0.02
**Diastolic *parameters***			
Early (E)/Atrial velocity ratio [n]	1.1 (0.8–1.4) [14]	1.1 (0.8–1.2) [47]	0.29
Early maximal diastolic velocity (E’), cm/sec [n]	10 (8–12) [18]	12 (10–15) [54]	0.07
E/E’ [n]	4.7 (3.1–5.4) [14]	4.1 (3.2–5.4) [47]	0.61
***General parameters***			
Pulmonary acceleration time (m/s^2^) [n]	8.2 (7.1-8.8) [10]	10.5 (7.1-12.5) [39]	0.25
Right ventricle/left ventricle diameter* [n]	0.81 (0.73–0.87) [15]	0.79 (0.65–0.89) [46]	0.37
**Left ventricular function**			
***Systolic parameters***			
Myocardial performance index [n]	0.58 (0.44–0.68) [18]	0.42 (0.38–0.52) [55]	< 0.01
Ejection fraction,% [n]	43 (37–53) [18]	55 (47–61) [55]	< 0.01
Global longitudinal strain,% [n]	−12 (−14—10) [18]	−14 (−18—10) [55]	0.09
Isovolumetric acceleration, m/sec [n]	1.5 (1.1–2.8) [17]	2.5 (1.7–4.1) [55]	0.01
Systolic maximal velocity, cm/sec [n]	7.5 (6.0–10.0) [18]	8.7 (7.0–10.0) [55]	0.21
***Diastolic parameters***			
Early (E)/atrial velocity ratio [n]	0.9 (0.7–1.2) [14]	1.0 (0.7–1.2) [54]	0.91
Early maximal diastolic velocity (E’), cm/sec [n]	8.0 (7.0–10.0) [18]	8.5 (6.5–11.0) [55]	0.62
E/E’ [n]	6.9 (5.7–10.1) [14]	8.2 (6.2–10.8) [54]	0.32
***General parameters***			
Cardiac index, L/min/m^2^ [n]	2.00 (1.63–2.92) [13]	2.57 (1.93–3.36) [49]	0.06
Eccentricity index [n]	1.00 (0.85–1.26) [15]	0.92 (0.81–1.05) [46]	0.23

*[n], number of patients for which this measure was available.*

*Basal diameters (endocardial to endocardial surface) obtained in the four-chamber view at the end of diastole.

### Association of Right Ventricular Myocardial Performance Index With Liberation Form Invasive Ventilation

**FIGURE 2:**
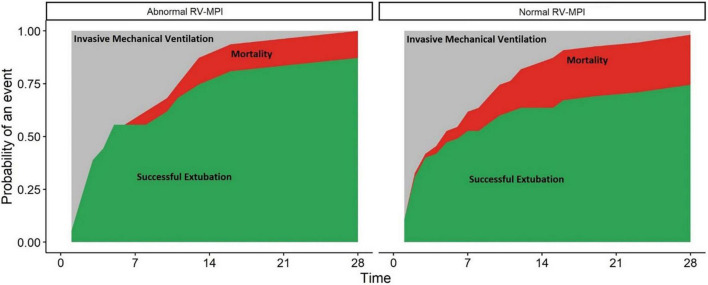
Abnormal right ventricular myocardial performance index (RV-MPI > 0.54) and normal RV-MPI (≤0.54) and cumulative incidence of outcomes. *X*-axis: Days since intubations. *Y*-axis: probability of an event (extubation or death) in the population. The two facets show the risk for patients with an abnormal RV-MPI **(left)** and normal RV-MPI **(right)**. Red areas represent patients who died. Green areas represent patients who were successfully extubated. Gray areas represent patients who remained invasively ventilated.

The RV-MPI, used as a continuous variable, was not associated with successful liberation from invasive ventilation before day 28 [HR, 2.2 (95% CI 0.47–10.6); *p* = 0.31]. RV-MPI > 0.54 was also not associated with a lower probability of successful liberation from mechanical ventilation [HR, 0.89 (95% CI 0.49–1.62); *p* = 0.72] (Figure 2).

### Association of Right Ventricular Myocardial Performance Index With Mortality

The RV-MPI was not associated with 28-day mortality [HR, 1.56 (95% CI 0.07–34.27); *p* = 0.78]. An RV-MPI > 0.54 was also not associated with mortality [HR, 2.1 (95% CI 0.46–9.17); *p* = 0.34] (Figure 2).

### Associations of Other Echocardiography-Derived Parameters for Right Ventricular Function With Outcomes

Other echocardiography-derived parameters for RV function were not associated with successful liberation from invasive ventilation before day 28 (Table 3).

**TABLE 3:** The association of right ventricular parameters obtained with transthoracic echocardiography with the probability of successful liberation from invasive ventilation and death at 28 days.

Variables	Hazard ratio (95% CI)	*P*-value
**Endpoint: Successful extubation**		
Myocardial performance index	2.2 (0.47–10.63)	0.30
Tricuspid annular plane systolic excretion	0.9 (0.95-1.04)	0.93
Systolic maximal velocity	1.0 (0.94-1.06)	0.91
Global longitudinal strain	1.1 (0.99-1.08)	0.10
Right ventricle/left ventricle diameter	0.6 (0.21-2.11)	0.48
**Endpoint: Mortality**		
Myocardial performance index	1.6 (0.07–34.27)	0.77
Tricuspid annular plane systolic excretion	1.1 (0.93-1.12)	0.59
Systolic maximal velocity	1.1 (0.87-1.09)	0.67
Global longitudinal strain	0.9 (0.86-1.01)	0.09
Right ventricle/left ventricle diameter	0.5 (0.22-14.33)	0.58

### Subgroup Analyses

PEEP levels were not different between patients with normal RV-MPI and those with abnormal RV-MPI (Table 4), and there was no evidence of moderation by PEEP of the associations of RV-MPI with outcome (*p* = 0.81). V_T_ was higher in patients with RV-MPI > 0.54 (Table 4), but there was no evidence of moderation by V_T_ of the association of RV-MPI with outcome (*p* = 0.35).

**TABLE 4:** Respiratory and hemodynamic variables at the time of transthoracic echocardiography according to normal or abnormal right ventricular myocardial performance index (RV-MPI).

Variables	Abnormal RV-MPI (*n* = 18)	Normal RV-MPI (*n* = 55)	*P*-value
**Respiration**			
Tidal volume, ml/kg PBW, median (IQR)	8.55 (7.47–9.72)	7.3 (5.7–8.6)	0.02
PEEP, cm H_2_O, median (IQR)	5 (5–7)	8 (1–8)	0.56
FiO_2_,%, median (IQR)	25 (22–33)	30 (25–35)	0.51
SpO_2_, median (IQR)	95 (94–96)	97 (94–98)	0.39
RR, breaths/min, median (IQR)	17 (14–21)	19 (15–24)	0.27
**Laboratory**			
pH, median (IQR)	7.43 (7.40–7.48)	7.44 (7.40–7.46)	0.97
PaCO_2_, kPa, median (IQR)	4.9 (4.21–5.75)	5.0 (4.5–5.4)	0.67
PaO_2_, kPa, median (IQR)	10.8 (10.2–11.7)	10.6 (9.7–11.9)	0.81
**Hemodynamics**			
Heart rate, mmHg, median (IQR)	90 (79–103)	80 (67–99)	0.18
Systolic blood pressure, mmHg, median (IQR)	118 (104–141)	130 (109–163)	0.29
Diastolic blood pressure, mmHg, median (IQR)	67 (57–79)	65 (56–72)	0.48
Mean arterial pressure, mmHg, median (IQR)	86 (73–92)	85 (76–102)	0.82

*PBW, per predicted body weight; PEEP, positive end expiratory pressure; FiO_2_, fraction inspired oxygen; SpO_2_, peripheral oxygen saturation; RR, respiratory rate; PaCO_2_, partial pressure of carbon dioxide in the arterial blood; PaO_2_, partial pressure of carbon dioxide in the arterial blood.*

## Discussion

The findings of this study can be summarized as follows: (1) RV-MPI is abnormal in a substantial number of patients who receive invasive ventilation for reasons other than ARDS; (2) in these patients, RV-MPI is neither associated with successful liberation from the ventilator within 28 days; (3) nor with 28-day mortality.

The findings of our study are in contrast to the results of one previous study (14). Indeed, in that study, RV-MPI was strongly associated with the duration of ventilation. Several difference between our study and that previous study should be mentioned, though. First, that study enrolled patients with ARDS, while we restricted the enrollment of patients not having ARDS. Second, and probably as a consequence of this, patients in the previous study were ventilated with higher PEEP than in our study. The results of this study add to our understanding of the association of RV-MPI with liberation of mechanical ventilation and mortality in critically ill patients, by showing that the prognostic value of RV-MPI may depend on the presence of ARDS and may be also the level of PEEP.

The findings of our study are in line with the results of several other studies (20–22) and one meta-analysis (23), Indeed, these investigations did not find an association of right ventricular dysfunction with successful liberation from invasive ventilation. Of note, associations of diastolic left ventricular function with successful liberation from invasive ventilation have been reported before (23). An abnormal right ventricular function could be associated with an abnormal systolic or diastolic left ventricular function (24–26). However, only left ventricular diastolic dysfunction, and not systolic dysfunction, has been found to have an association with successful extubation (23), and in our cohort, we did find only systolic, and not diastolic dysfunction of the left ventricle.

Right ventricular myocardial performance index is, at least in part, preload-dependent, and the size of V_T_ and level of PEEP could affect the preload of the right ventricle in invasively ventilated patients. In our cohort, patients were ventilated with higher or lower V_T_ (15), and with higher or lower PEEP (16), as per the study protocols of the two parent studies. Of note, several patients treated with V_T_ near to 10 ml/kg (PBW) which can cause significant instant preload variations (27) affecting, in theory, RV-MPI measurement reliability and, consequently, its association with the outcome. In this relatively small *post hoc* study, we could not clearly establish the relationship between V_T_ and RV dysfunction, and it cannot be excluded that V_T_ actually affects RV-MPI. One potential weakness of our approach is that there could be an interaction between the intervention being tested in the original trial(s) and the outcome of the secondary analysis. We cannot exclude that ventilation with high V_T_ does not affect the reliability of RV-MPI to evaluate right heart function.

The results of this study can be used to decide on whether RV-MPI should be monitored with transthoracic echocardiography in invasively ventilated patients without ARDS. One could hypothesize that right ventricular dysfunction is in part caused by higher intrathoracic pressures, as patients randomized to ventilation with higher V_T_ and patients randomized to ventilation with higher PEEP more often had an abnormal RV-MPI. While we show that RV-MPI has no predictive validity, we cannot exclude that RV-MPI may be useful in guiding fluid and inotrope therapy in these patients. While RV-MPI seems a relatively easy to collect index, in 8 out of 81 patients, we were not able to capture it. However, other parameters are usually more difficult to collect—-for instance, right ventricular global longitudinal strain, another parameter for right ventricular function could not be measured in more than a quarter of these patients.

The strength of this study was the systematic evaluation of the prognostic validity of RV function in a homogeneous population of critically ill patients without ARDS. Patients were examined soon after the start of invasive ventilation, thereby reducing the risk of the effects of other strategies, as well as bias by left truncation. We also excluded patients with pre-existing heart failure. Echocardiographic parameters were evaluated in a blind fashion, and only in a small portion of patients, the RV-MPI could not be collected.

This study also has limitations. First, although the sample size was larger than that in most other studies on this topic, the confidence intervals were wide and repeating this study in a larger cohort of patients would likely result in a more precise estimate of effect. Seen the lack of previous studies on associations of RV-MPI with outcome in this specific group of critically ill patients, we were not able to perform a proper sample size calculation. Nevertheless, given that the observed difference in mortality was opposite to the hypothesis, the likelihood that a larger sample size would provide an opposite result (a higher mortality in patients with a more abnormal RV function) is minimal. Second, we did not analyze inter-observer and intra-observer variability of MPI measurement; however, all measurements were taken blindly for the ventilator settings. Third, patients were evaluated only one time in the acute phase, and we cannot exclude the possibility that some patients developed right ventricular dysfunction at later timepoints in the course of their disease or in response to certain treatments, like the administration of fluid, or the use of inotropes and vasopressors.

## Conclusion

In this *post hoc* analysis of two studies in invasively ventilated critically ill patients without ARDS, an abnormal RV-MPI indicative of RV dysfunction was prevalent, but was not associated with successful liberation from invasive ventilation or death. The association between RV function should be further studied in prospective investigations that have a larger sample size, possibly focusing on patients with a higher likelihood of extubation failure specifically.

## Data Availability Statement

The raw data supporting the conclusions of this article will be made available by the authors, without undue reservation.

## Ethics Statement

The studies involving human participants were reviewed and approved by the AMC Review Board. The patients/participants provided their written informed consent to participate in this study.

## Author Contributions

CP, LB, and MS contributed to study conception and design, data analysis, and interpretation, drafted the manuscript, and approved the submitted version of the manuscript. AG, FS, TC, WL, and FP revised the manuscript for critical content and approved the submitted version of the manuscript. PReVENT– and the RELAx–investigators performed the parent studies and by that made it possible to design and to perform this study. All authors contributed to the article and approved the submitted version.

## Conflict of Interest

The authors declare that the research was conducted in the absence of any commercial or financial relationships that could be construed as a potential conflict of interest.

## Publisher’s Note

All claims expressed in this article are solely those of the authors and do not necessarily represent those of their affiliated organizations, or those of the publisher, the editors and the reviewers. Any product that may be evaluated in this article, or claim that may be made by its manufacturer, is not guaranteed or endorsed by the publisher.

## Footnotes

1
www.r-project.org
